# Cigarette Smoke Extract Stimulates MMP-2 Production in Nasal Fibroblasts via ROS/PI3K, Akt, and NF-κB Signaling Pathways

**DOI:** 10.3390/antiox9080739

**Published:** 2020-08-12

**Authors:** Joo-Hoo Park, Jae-Min Shin, Hyun-Woo Yang, Tae Hoon Kim, Seung Hoon Lee, Heung-Man Lee, Jae-Gu Cho, Il-Ho Park

**Affiliations:** 1 Upper Airway Chronic inflammatory Diseases Laboratory, Korea University College of Medicine, Seoul 05505, Korea; pjh52763@naver.com (J.-H.P.); shinjm0601@hanmail.net (J.-M.S.); yhw444@gmail.com (H.-W.Y.); 2 Department of Otorhinolaryngology-Head and Neck Surgery, Korea University College of medicine, Seoul 05505, Korea; doctorth@korea.ac.kr (T.H.K.); shleeent@korea.ac.kr (S.H.L.); lhman@korea.ac.kr (H.-M.L.)

**Keywords:** cigarette smoke extract, nasal fibroblasts, tissue inhibitor of metalloproteinases, matrix metalloproteinase, steroids

## Abstract

Cigarette smoke exposure has been shown to be associated with chronic rhinosinusitis and tissue remodeling. The present study aimed to investigate the effects of cigarette smoke extract (CSE) on matrix metalloproteinase (MMP) and tissue inhibitor of metalloproteinase (TIMP) production in nasal fibroblasts and to determine the underlying molecular mechanisms. Primary nasal fibroblasts from six patients were isolated and cultured. After the exposure of fibroblasts to CSE, the expression levels of MMP-2, MMP-9, TIMP-1, and TIMP-2 were measured by real-time PCR, ELISA, and immunofluorescence staining. The enzymatic activities of MMP-2 and MMP-9 were measured by gelatin zymography. Reactive oxygen species (ROS) production was analyzed using dichloro-dihydro-fluorescein diacetate and Amplex Red assays. PI3K/Akt phosphorylation and NF-κB activation were determined by Western blotting and luciferase assay. CSE significantly increased MMP-2 expression and inhibited TIMP-2 expression but did not affect MMP-9 and TIMP-1 expression. Furthermore, CSE significantly induced ROS production. However, treatment with ROS scavengers, specific PI3K/Akt inhibitors, NF-κB inhibitor, and glucocorticosteroids significantly decreased MMP-2 expression and increased TIMP-2 expression. Our results suggest that steroids inhibit CSE-regulated MMP-2 and TIMP-2 production and activation through the ROS/ PI3K, Akt, and NF-κB signaling pathways in nasal fibroblasts. CSE may contribute to the pathogenesis of chronic rhinosinusitis by regulating MMP-2 and TIMP-2 expression.

## 1. Introduction

Chronic rhinosinusitis (CRS) is a nasal inflammatory disease with symptoms of nasal discharge/postnasal drip, nasal congestion, sinus pain/pressure, and anosmia/hyposmia lasting for at least 12 weeks. CRS is one of the most frequent chronic diseases in humans and consequently has an important socio-economic impact. Its influence on patient quality of life is even more detrimental than that of congestive heart failure, chronic obstructive pulmonary disease, and back pain [1]. CRS is a multifactorial disorder, and its pathogenesis involves interactions between environmental insults, infectious loading, and genetic predisposition. Among environmental factors, inhaled pollutants, including cigarette smoke, may play a significant role in CRS, which is a characteristic of chronic inflammatory upper airway disease [2].

Tissue remodeling is an energetic process that results in both production and degradation of the extracellular matrix (ECM) and is an important aspect in the pathogenesis of chronic inflammatory diseases in many organs. In airways, tissue remodeling is characterized by loss of epithelial integrity, goblet cell metaplasia, excessive ECM deposition, hyperplasia of mucosal cells, and basement membrane thickening. The differentiation of fibroblasts to myofibroblast is a key event in physiological and pathological tissue remodeling. Myofibroblasts are a source of matrix metalloproteinases (MMPs) and tissue inhibitors of MMPs (TIMPs) [3]. Like other chronic inflammatory diseases of the airway, tissue remodeling is present in CRS, and obvious remodeling features differentiate the various CRS subgroups [4]. Several factors, such as MMPs, ECM, and TGF-β, are related with remodeling. Specifically, MMP-2 and MMP-9 have been associated with airway inflammatory diseases [5]. MMPs are a large family of enzymes with zinc-binding catalytic domains and are involved in the degradation of ECM components. Their extracellular activities are regulated by TIMPs. MMPs play a crucial role in various physiological processes, including tissue remodeling [6]. MMP-2 (gelatinase A) and MMP-9 (gelatinase B) are involved in ECM remodeling, which is associated with upper airway remodeling [7]. MMP-2 and MMP-9 are the principal fibroblast-derived proteinases capable of degrading substrates, including collagen, gelatin, elastin, and fibronectin.

Chronic exposure to cigarette smoke is known to be the cause of several inflammatory diseases including asthma, chronic obstructive pulmonary disease, and CRS. Cigarette smoke extract (CSE) exerts harmful effects on processes, including cell viability, adhesion, migration, and myofibroblast differentiation, in the airway. Tissue remodeling is a pathologic process believed to be related with cigarette smoke. It was previously shown that smoke can directly induce remodeling without any need for exogenous inflammatory cells in the airway [8].

CSE contains many toxic and carcinogenic chemicals, as well as unsuitable free radical that enhance reactive oxygen species (ROS) production leading to oxidative stress. ROS is known to be higher expressed in patients with CRS was higher than in control subject [9]. It damages proteins, lipids, and DNA that plays an important roles in cellular process involved in the generation and development processes of nasal polyps [10]. Airway inflammation, airway hyper-responsiveness, tissue injury, and remodeling can be induced by excessive ROS production in epithelial cells, fibroblasts and severer inflammatory cells [11,12]. Increased ROS can mediate activation of AKT and NF-κB [13]. PI3K and Akt proteins in CRS were higher than those in the control subjects [14]. The PI3K/Akt signaling pathway is an important cellular signaling that is involved in cell growth, proliferation, apoptosis, metabolism, angiogenesis, metastasis and the cellular defiance against inflammatory stimuli. Akt cascade is known to mediate ECM proteolysis [15]. NF-κB is a transcriptional factor which plays a central role in diverse cellular processes, including inflammation and immune response in airway diseases. CSE modulates variety molecular mechanisms such as ROS, PI3K/AKT and NF-κB. However, the precise mechanisms by which cigarette smoke affects airway structure and function are still under investigation.

We hypothesized that CSE-induced tissue remodeling in the upper airway is related with an imbalance between MMPs and TIMPs from nasal fibroblasts. In the present study, we aimed to determine the effects of CSE on the expression of MMPs and TIMPS and the underlying molecular mechanisms in nasal fibroblasts. We also examined the involvement of ROS, AKT, and NF-κB signaling pathways, which are known to be closely related with CSE-induced inflammation in several diseases, in these mechanisms. Additionally, we aimed to determine the effect of glucocorticoids, the first line of treatment for CRS, on the CSE-induced imbalance between MMPs/TIMPs in nasal fibroblasts.

## 2. Materials and Methods

### 2.1. Preparation of CSE

CSE was obtained from 3R4F research cigarettes (University of Kentucky, Lexington, KY, USA), with each cigarette containing 0.60 mg of nicotine and 8.0 mg of tar, for use in all experiments. CSE was prepared by bubbling smoke from cigarettes into 15 mL of serum-free Dulbecco’s modified Eagle’s medium (DMEM; Invitrogen, Grand Island, NY, USA) at a rate of 1 cigarette/min using a modification of the method developed by Carp and Janoff [16]. The CSE solution is defined as 100% CSE and diluted with DMEM in the following experiments. CSE was standardized by measuring of the absorbance at 320 nm confirmed that the prepared CSE was reproducible, and various CSE preparations showed few differences. Freshly prepared CSE was used immediately in all the experiments.

### 2.2. Patients and Tissue Collection

Sinus tissue explants were collected during surgery from patients with blowout fracture (*n* = 6) at the Department of Otorhinolaryngology at Korea University Guro Hospital, Korea. All patients had no history of smoking, allergies, asthma, or aspirin sensitivity, and they were not treated with any antibiotics or oral or topical steroids for at least 4 weeks before surgery. Written consent was obtained from all patients, and the study was approved by the Ethics Committee of the Faculty of Medicine, Korea University, Korea.

### 2.3. Nasal Fibroblast Cultures

To obtain nasal fibroblasts, cells were cultured in DMEM with 10% heat-inactivated fetal bovine serum (FBS), 1% (*v/v*) 10,000 U/mL penicillin, and 10,000 μg/mL streptomycin (Invitrogen) in a humidified incubator under 5% CO_2_ at 37 °C. Experiments were performed using cells at 80% confluence. Before treatment of agents, the cells were starved in serum-free media for 12 h. The purity of obtained human nasal fibroblasts was confirmed microscopically based on the characteristic spindle-like cell phenotype. Approximately 95% of cells in cultured nasal fibroblasts were positive for vimentin and Thy-1, which were used as fibroblast markers, and negative for E-cadherin, which was used as an epithelial cell marker. The nasal fibroblasts were cultured for four passages [17].

### 2.4. Real-Time PCR

The nasal fibroblasts were incubated with CSE for 12 h after pre-treatment of ROS scavengers (NAC, ebselen and DPI) PI3K/Akt inhibitor (LY294002) and NF-κB inhibitor (BAY11-7082) for 1 h. RNAs were extracted from nasal fibroblasts using TRIzol reagent (Invitrogen, Carlsbad, CA, USA). The total amount of RNA was determined using NanoDrop 2000 (Thermo Fisher Scientific Inc., Wilmington, DE, USA). Synthesis of cDNA was performed with 1 μg of total RNA using the Maxime RT PreMix cDNA Kit (iNtRON Biotechnology, Sungnam, Korea). The expression levels of mRNAs were analyzed using Quantstudio3 (Applied Biosystems, Foster City, CA, USA) and Power SYBR Green PCR Master Mix (Applied Biosystems). Real-time PCR analysis was performed to evaluate the expression levels of *MMP-2, MMP-9, TIMP-1,* and *TMIP-2* and that of *GAPDH*, which was used as a housekeeping gene. The primer sequences are shown in Table 1. The results were normalized by *GAPDH* mRNA expression and are shown as the fold ratio over the expression of the control group.

### 2.5. Gelatin Zymography

Nasal fibroblasts were exposed to CSE for 72 h after pretreatment with ROS scavengers, PI3K/Akt inhibitor and NF-κB inhibitor for 1 h. Aliquots of fibroblast-conditioned medium (10 µL) were analyzed using gelatin zymography for MMP-2 and MMP-9 in 1 mg/mL gelatin-10% polyacrylamide gels. Following electrophoresis, the gels were washed twice with 2.5% Triton X-100 for 30 min while shaking to remove sodium dodecyl sulfate and renature MMP-2 and MMP-9 in the gels. Renatured gels were incubated in developing buffer containing 50 mM Tris-HCl (pH 7.5), 200 mM NaCl, 5 mM CaCl_2_, and 0.02% Brij-35 overnight at 37 °C. Gels were stained with 0.25% Coomassie brilliant blue G-250 (50% methanol, 10% acetic acid) and destained using destaining solution (50% methanol, 10% acetic acid). Proteinase activity was observed as cleared (unstained) regions on the gels. Finally, the gels were dried for 2 h using a gel dryer (Bio-Rad, Hercules, CA, USA).

### 2.6. Enzyme-Linked Immunosorbent Assay (ELISA)

MMP-2, MMP-9, TIMP-1, and TMIP-2 concentrations in the culture media were determined using ELISA (R&D systems, Minneapolis, MN, USA). Nasal fibroblasts were exposed to CSE for 72 h after pretreatment with ROS scavengers, PI3K/Akt inhibitor and NF-κB inhibitor for 1 h. Standards and samples were added and incubated at room temperature for 2 h. After 3 washes, MMP-2, MMP-9, TIMP-1, or TMIP-2 conjugate was added to the wells for 2 h at room temperature. The reaction was stopped with a stop solution, and the product was quantified at 450 nm using a microplate reader (Bio-Rad).

### 2.7. Western Blotting Analysis

Nasal fibroblasts were treated with CSE for 72 h. A total of 5 × 10^5^ fibroblasts were lysed in PRO-PREP^TM^ protein extraction solution (iNtRON Biotechnology) and stored overnight at −20 °C. Cell debris was removed from the lysates by centrifugation at 13,000× *g* for 30 min at 4 °C. Total protein concentration was determined using the Bradford assay (Bio-Rad). An equal quantity of protein from samples (30 µg) were separated using 12% sodium dodecyl sulfate polyacrylamide gel electrophoresis, transferred to 0.45 μm polyvinyl difluoride membranes, (Millipore Inc., Billerica, MA, USA), and analyzed separately. Membranes were blocked with 5% skim milk at room temperature for 60 min, rinsed three times with Tris-buffered saline containing Tween-20, and treated with the following primary antibodies: polyclonal p-PI3K (1:1000, #4228, Cell Signaling Technology, Danvers, MA, USA), total-PI3K (1:1000, #4292, Cell Signaling Technology), p-Akt (1:1000, #9271, Cell Signaling Technology), total-Akt (1:1000, #9272, Cell Signaling Technology), p-p65 (1:1000, sc-136548, Santa Cruz Biotechnology Inc., Santa Cruz, CA, USA), total-p65 (1:1000, sc-8008, Santa Cruz Biotechnology Inc.) and β-actin (1:10000, sc-47778, Santa Cruz Biotechnology Inc.). Bands were visualized using horseradish peroxidase-conjugated secondary antibodies and an enhanced chemiluminescence system (Pierce, Rockford, IL, USA).

### 2.8. Immunofluorescent Staining

Nasal fibroblasts were placed onto coverslips and treated with CSE for 72 h. Fibroblasts were fixed with 4% paraformaldehyde and then permeated with 0.2% Triton X-100 in 1% FBS for 10 min at room temperature. After treating coverslips with 5% BSA to block for 1 h at room temperature, fibroblasts were incubated overnight at 4 °C with anti-MMP-2, anti-MMP-9, anti-TIMP-1, or anti-TIMP-2 antibodies (Santa Cruz Biotechnology, Inc. CA, USA). Goat anti-mouse Alexa 488 (Invitrogen) secondary antibody was also added to fibroblasts and incubated. Lastly, 4′-6-diamidino-2-phenylindole (DAPI) was applied for counterstaining. The stained normal fibroblasts were subsequently maintained on a confocal laser scanning microscope (LSM700, Zeiss, Oberkochen, Germany).

### 2.9. Measurement of ROS Produced

Intracellular ROS production was determined using a fluorescent probe, 2, 7-dichlorodihydrofluorescein diacetate (Molecular Probes, Inc., Eugene, OR, USA). Cells were pretreated with N-acetyl-L-cysteine (NAC, 5 mM), ebselen (10 μM), or diphenyleneiodonium (DPI, 2 μM) for 1 h and then stimulated with CSE for 12 h. After that, CSE-stimulated cells were suspended in serum-free culture medium with H2DCF-DA (10 μM) for 30 min. Cellular fluorescence was measured using fluorescence microscopy (IX71; Olympus, Life Science Solutions, Tokyo, Japan) to observe ROS production. The H_2_O_2_ production was measured using an Amplex Red Hydrogen Peroxide/Peroxidase Assay Kit (Thermo Fisher Scientific Inc.), according to the manufacturer’s instructions. Nasal fibroblasts were pretreated with NAC, ebselen, or DPI. Prior to CSE stimulation, the cell lysate (50 μL) was treated with a working solution of 100 μM Amplex Red reagent (Thermo Fisher Scientific Inc.) and 0.2 U/mL horseradish peroxidase. After incubation for 30 min at 37 °C, fluorescence was measured at 560 nm on a microplate reader (Bio-Rad).

### 2.10. Statistical Data Analysis

For all outcomes, data were obtained in triplicate from at least three separate experiments. Data are shown as mean ± SEM of three different experiments in triplicate. All significant differences between controls and examined samples were analyzed by one-way analysis of variance followed by Tukey’s test (GraphPad Prism version 5; GraphPad Software, San Diego, CA, USA). Significance was considered at a 95% confidence level. P-values below 0.05 were considered statistically significant.

## 3. Results

### 3.1. Effects of CSE on MMP and TIMP Production in Nasal Fibroblasts

**Figure 1:**
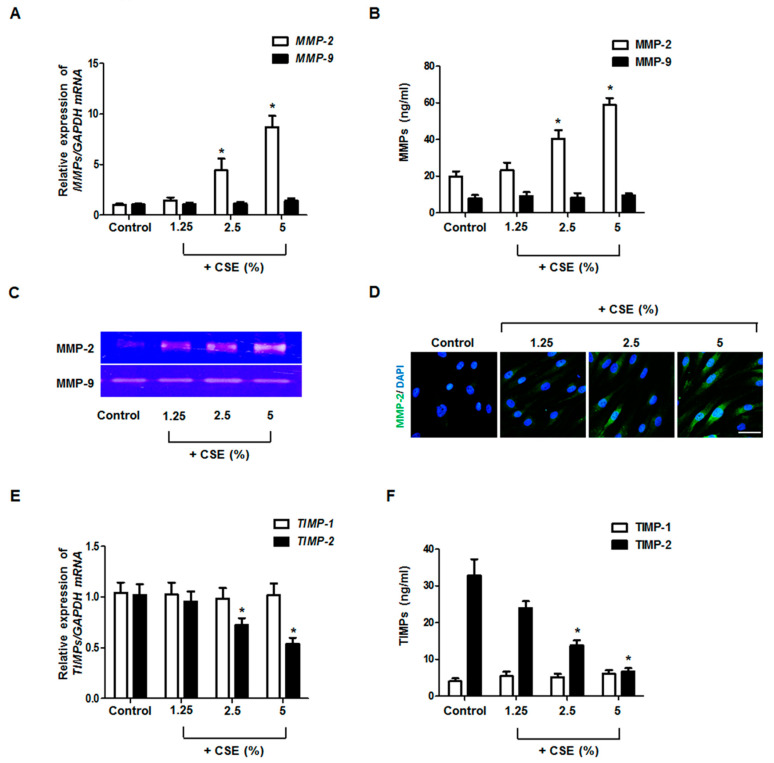
Effect of CSE on MMP and TIMP expression in nasal fibroblasts. After treatment of nasal fibroblasts with 5% CSE, the mRNA levels of *MMPs* and *TIMPs* were measured using real-time PCR (**A**,**E**), and MMP and TIMP protein expression levels were determined using ELISA (**B**,**F**). The enzymatic activities of MMP-2 and MMP-9 were measured by gelatin zymography (**C**). The expression and localization of MMP-2 protein (green) were observed using immunofluorescence staining (**D**). * *p* < 0.05 vs. control. Scale bar = 100 μm.

To determine whether CSE regulates the production of MMPs and TIMPs, we treated fibroblasts with CSE at various concentrations (0–5%). CSE increased MMP-2 expression dose-dependently but did not affect the expression of MMP-9 mRNA and protein (Figure 1A,B). Gelatin zymography, used to evaluate enzymatic activity, showed that only MMP-2 activity was increased by CSE treatment (Figure 1C). The stimulatory effect of CSE on MMP-2 protein was also confirmed by immunofluorescence staining (Figure 1D). CSE decreased TIMP-2 expression but did not affect the expression of TIMP-1 (Figure 1E,F). These results indicate that CSE induced MMP-2 production and decreased TIMP-2 production in nasal fibroblasts.

### 3.2. Role of ROS in MMP and TIMP Production in CSE-Stimulated Nasal Fibroblasts

**Figure 2:**
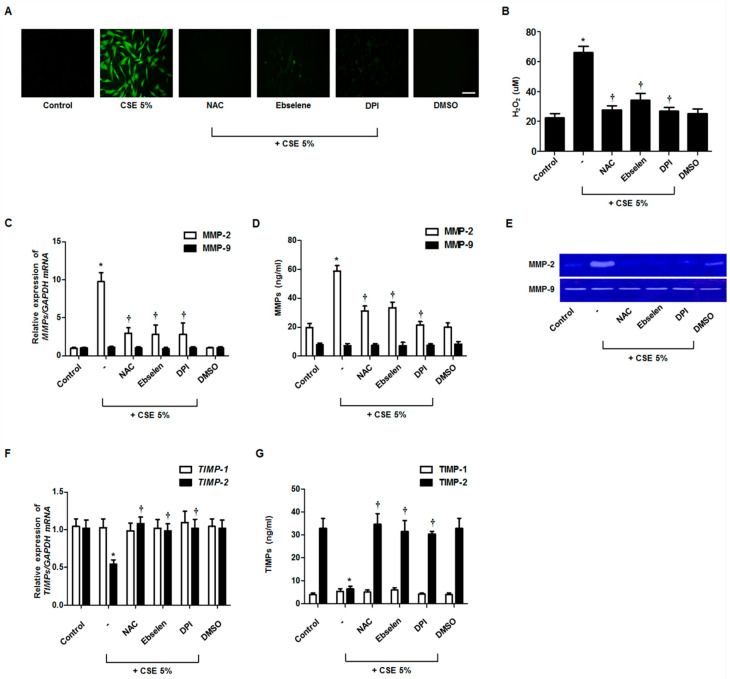
Effects of ROS on CSE-regulated MMP and TIMP expression in nasal fibroblasts. Total ROS and mitochondrial superoxides were quantified using the 2,7-dichlorofluorescein diacetate assay and Amplex Red assay (**A**,**B**). Fibroblasts were pretreated with NAC (1 mM), ebselen (10 μM), and DPI (2 μM) before being treated with CSE (5%). The expression levels of MMP and TIMP mRNAs were determined by real-time PCR (**C**,**F**). MMP and TIMP protein levels were determined using ELISA (**D**,**G**). The enzymatic activities of MMP-2 and MMP-9 were measured by gelatin zymography (**E**). * *p* < 0.05 vs. control; ^†^ *p* < 0.05 vs. CSE only. Scale bar = 50 μm.

To investigate the role of ROS in MMP-2 and TIMP-2 production, nasal fibroblasts were pretreated with ROS scavengers 1 h before CSE treatment. In DCFH-DA and Amplex Red staining assays, CSE treatment induced the production of ROS and hydrogen peroxide; however, such production was blocked by the addition of NAC, ebselen, or DPI (Figure 2A,B). Next, the effects of ROS scavengers on mRNA and protein expression were measured using real-time PCR and western blotting. Pretreatment with ROS scavengers blocked the stimulatory effects of CSE on MMP-2 expression (Figure 2C,D). This result was also observed through gelatin zymography (Figure 2E). Inversely, pretreatment with antioxidants blocked the inhibitory effect of CSE on TIMP-2 expression (Figure 2F,G). MMP-9 and TIMP-1 production was not affected by ROS scavengers in nasal fibroblasts.

### 3.3. Involvement of the PI3K/Akt Cascade in MMP and TIMP Production in CSE-Stimulated Nasal Fibroblasts

**Figure 3:**
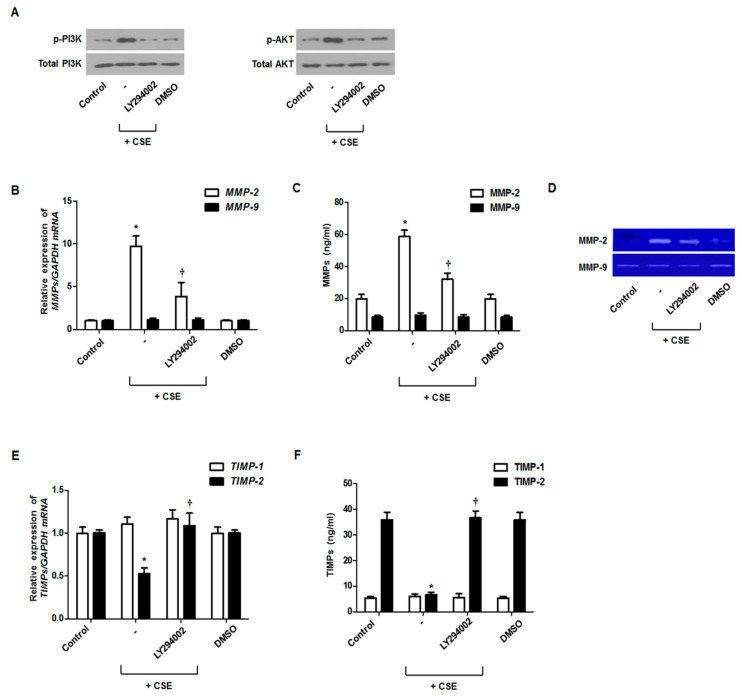
Regulation of PI3K/Akt signaling pathways with CSE regulated MMP and TIMP expression. Nasal fibroblasts were pretreated with LY294002 (PI3K/Akt inhibitor) before treatment with 5% CSE. Levels of phosphorylated (p)-PI3K and p-Akt were determined using western blotting (**A**). The expression levels of MMP and TIMP mRNAs were determined by real-time PCR (**B**,**E**). MMP and TIMP protein levels were determined using ELISA (**C**,**F**). The enzymatic activities of MMP-2 and MMP-9 were measured by gelatin zymography (**D**). * *p* < 0.05 vs. control; ^†^ *p* < 0.05 vs. CSE only.

The fibroblasts were treated with CSE and a PI3K/Akt inhibitor to confirm whether a PI3K/Akt pathway is involved in the expression of MMP-2 and TIMP-2. CSE induced PI3K/Akt phosphorylation, which was inhibited by the PI3K/Akt inhibitor (LY294002) (Figure 3A). Next, we confirmed that the PI3K/Akt inhibitor suppressed MMP-2 mRNA and protein expression and enzymatic activation, which are induced by CSE in nasal fibroblasts (Figure 3B–D). On the contrary, TIMP-2 mRNA and protein expression was significantly increased by treatment with the PI3K/Akt inhibitor in CSE-stimulated nasal fibroblasts (Figure 3E,F). Additionally, we confirmed that ROS inhibition suppressed the activation of PI3K/AKT in nasal fibroblasts (data not shown). Therefore, we could assume that the signaling pathway associated with fibroblast activation under oxidative stress is attributable in part to the activation of the PI3K and AKT signaling pathways.

### 3.4. Effect of CSE on NF-κB Activation for MMP and TIMP Production

**Figure 4:**
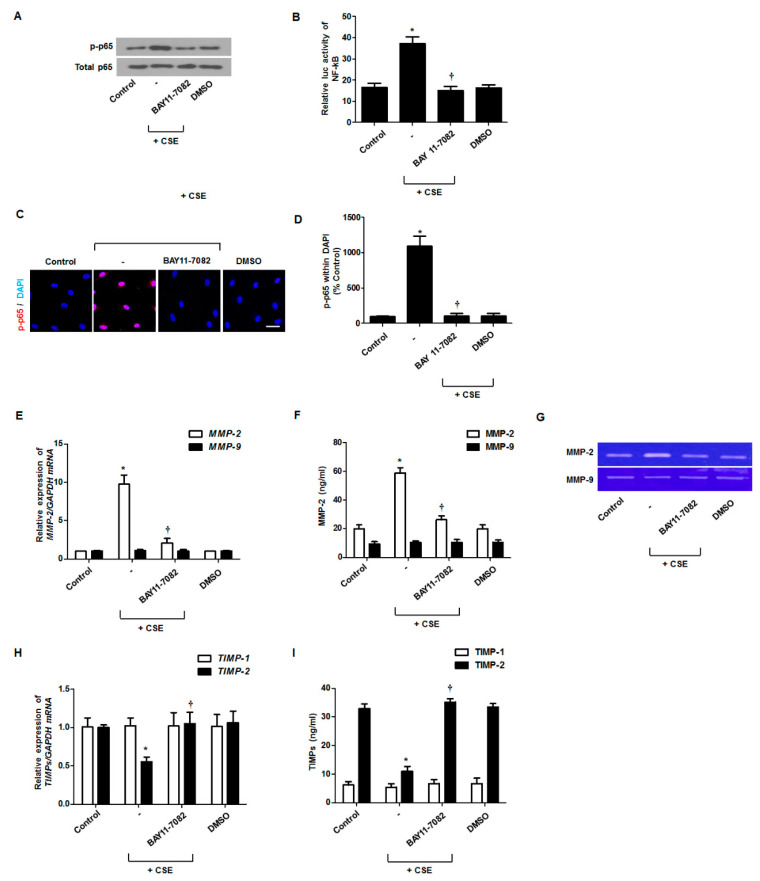
Effects of NF-κB activation on CSE-regulated MMP and TIMP levels in nasal fibroblasts. Nasal fibroblasts were pretreated with an NF-κB inhibitor (BAY 11-7082) before treatment with 5% CSE. Phospho (p)-p65 was measured using western blotting to determine NF-κB activation (**A**). NF-κB transcriptional activation was measured using the luciferase assay (**B**). After stimulation with CSE, the translocation of p-p65 protein (red) was observed by immunofluorescent staining. Magnification, ×400 (**C**,**D**). Nuclei were stained using DAPI (blue). The expression levels of MMP and TIMP mRNAs were determined by real-time PCR (**E**,**F**). The secretion levels of MMP and TIMP proteins were determined using ELISA (**H**,**I**). The enzymatic activities of MMP-2 and MMP-9 were measured by gelatin zymography (**G**). * *p* < 0.05 vs. control; ^†^ *p* < 0.05 vs. CSE only. Each experiment was performed three biological replicates. Scale bar = 100 μm.

The activated NF-κB enhanced MMP-2 expression in airway. To determine whether NF-κB activation is involved in MMP-2 and TIMP-2 expression, fibroblasts were pretreated with an NF-κB inhibitor (BAY11-7082) and then stimulated with CSE. Phosphorylated p65, a subunit of NF-κB, was induced by CSE treatment and inhibited by treatment with the NF-κB inhibitor (Figure 4A). NF-κB transcriptional activity, assessed by a luciferase reporter, was increased by CSE and then inhibited by the NF-κB inhibitor (Figure 4B). Immunocytochemical staining showed that CSE induced the translocation of p-p65 to the nucleus and that this was blocked by the NF-κB inhibitor (Figure 4C,D). Treatment with the NF-κB inhibitor inhibited MMP-2 mRNA and protein expression and activation of enzymatic capacity, which were stimulated by CSE (Figure 4E–G), and reversed the change in TIMP-2 mRNA and protein expression that had been inhibited by CSE treatment (Figure 4H,I). MMP-9 and TIMP-1 production was not affected by the NF-κB inhibitor in nasal fibroblasts.

### 3.5. Effect of Steroids on CSE-Regulated MMP and TIMP Production

**Figure 5:**
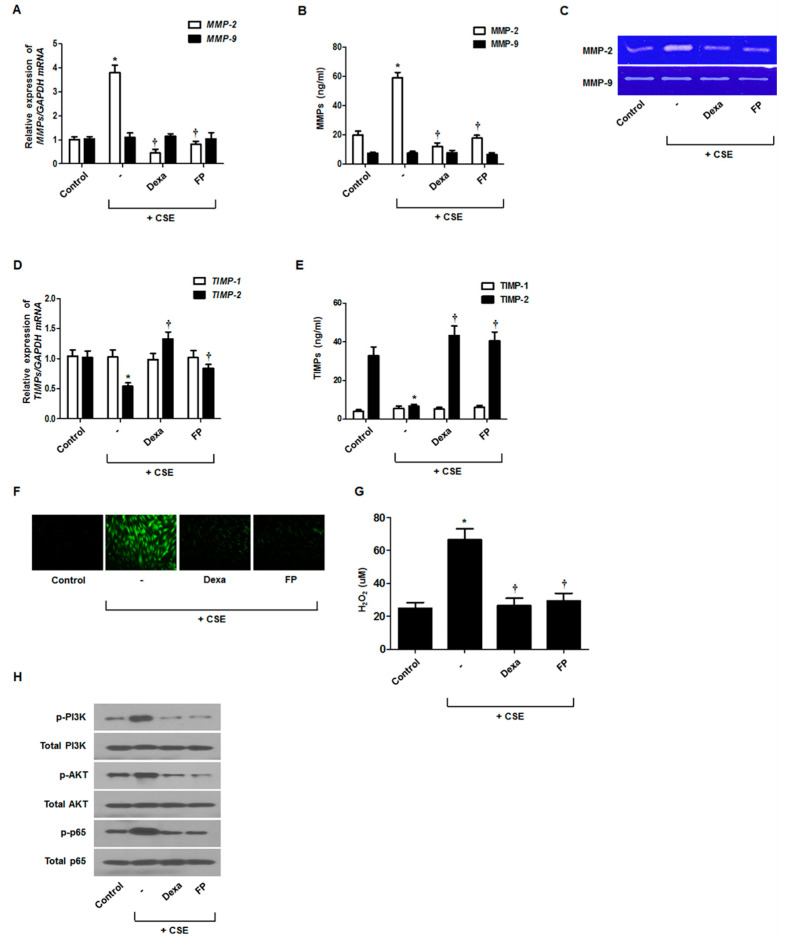
Effects of steroids on CSE-regulated MMP and TIMP levels in nasal fibroblasts. Nasal fibroblasts were pretreated with or without dexamethasone (Dexa, 2.5 μM) and fluticasone propionate (FP, 2.5 μM) before treatment with 5% CSE. The expression levels of MMP and TIMP mRNAs were determined by real-time PCR (**A**,**D**). MMP and TIMP protein secretion levels were determined using ELISA (**B**,**E**). The enzymatic activities of MMP-2 and MMP-9 were measured by gelatin zymography (**C**). Total ROS production and mitochondrial superoxides were quantified using the 2,7-dichlorofluorescein diacetate assay and Amplex Red assay (**F**,**G**). Levels of phosphorylated p-PI3K, p-Akt, and p-p65 were determined using western blotting (**H**). * *p* < 0.05 vs. control; ^†^ *p* < 0.05 vs. CSE only. Scale bar = 50 μm.

Dexamethasone (Dex) and fluticasone propionate (FP) are potent synthetic corticosteroids that are widely used as anti-inflammatory agents to treat respiratory diseases [18]. To assess whether steroids inhibited CSE-regulated MMP and TIMP production, fibroblasts were pretreated with dexamethasone or fluticasone propionate and then stimulated with CSE. MMP-2 expression was increased by CSE treatment and suppressed by steroids, whereas the opposite pattern was observed for TIMP-2 mRNA (Figure 5A,D) and protein levels (Figure 5B,E). Gelatin zymography showed similar patterns for MMP-2 (Figure 5C). Steroids did not affect MMP-9 and TIMP1 expression. Steroids significantly decreased ROS production, PI3K/Akt phosphorylation, and NF-κB activation in CSE-stimulated nasal fibroblasts (Figure 5F–H). These results suggested that steroids could regulate MMP-2 and TIMP-2 expression by blocking the ROS/PI3K/Akt and NF-κB signaling pathways in fibroblasts.

## 4. Discussion

The present study showed that CSE induced MMP-2 expression and decreased TIMP-2 expression but did not affect MMP-9 and TIMP-1 expression in nasal fibroblasts. CSE exposure induced ROS production. Treatment with ROS scavengers, such as NAC, ebselen, and DPI, suppressed CSE-regulated MMP-2 and TIMP-2 expression. Additionally, CSE induced PI3K/AKT phosphorylation and NF-κB activation. When CSE-stimulated nasal fibroblasts were treated with PI3K/AKT and NF-κB inhibitors, the CSE-mediated regulation of MMP-2 and TIMP-2 expression was suppressed in nasal fibroblasts. These data indicated that CSE induces MMP-2 expression and inhibits TIMP-2 expression via ROS, PI3K/AKT, and NF-κB signaling pathways in nasal fibroblasts.

Histopathological changes in the nasal mucosa of smokers are reported to differ from those in the nasal mucosa in nonsmokers [19]. In several studies, CSE exposure in airways increased inflammatory responses and tissue remodeling to aggravate chronic upper respiratory inflammation and diseases, such as CRS [2,20,21]. In CRS, histomorphological changes, including epithelial cell hyperplasia, basement membrane thickening, and ECM accumulation, occur in the respiratory system, leading to tissue remodeling [22]. An imbalance in MMPs and TIMPs, which are important factors involved in ECM homeostasis, leads to tissue remodeling [23]. Particularly, gelatinases, MMP-2, and MMP-9 are known to degrade almost all basement membrane components, including collagens, laminins, and gelatins, in tissue remodeling [24]. Bachert et al. showed that MMP-2 and MMP-9 expression was significantly enhanced in CRS patients compared to that in healthy controls [25]. We have previously reported that MMP-2 expression was increased in TGF-β1-stimulated nasal polyp-derived fibroblasts [26]. The present study demonstrated that CSE induces MMP-2 expression and inhibits TIMP-2 expression in nasal fibroblasts.

CSE contains high concentrations of oxidants that induce ROS, which play a significant role in the pathogenesis of diseases, such as CRS [27]. In particular, CSE-induced ROS may promote tissue remodeling. Fordham et al. showed that ROS were increased in the epithelia of patients with CRS, and exposure to CSE increased ROS in nasal tissues [27]. Thus, we showed that CSE induced an imbalance in MMP-2 and TIMP-2 through ROS in nasal fibroblasts. Additionally, ROS scavengers, including NAC, ebselen, and DPI, inhibited not only the imbalance in MMP-2 and TIMP-2 production, but also PI3K/Akt phosphorylation and NF-κB activation in CSE-stimulated nasal fibroblasts. PI3K/Akt phosphorylation and NF-κB activation are known to occur in inflammatory response progression [28]. Previous studies suggested that the PI3K/Akt pathway regulates various signaling pathways that lead to NF-κB activation [29]. CSE also caused PI3K/Akt phosphorylation and NF-κB activation in these signaling pathways in lung fibroblasts [30,31]. Intracellular signaling pathways, such as PI3K/Akt and NF-κB, have been shown to modulate MMP-2 and MMP-9 expression in lung tissues [32]. In our study, the inhibition of PI3K/Akt and NF-κB significantly suppressed CSE-enhanced MMP-2 expression and inhibited TIMP-2 expression in nasal fibroblasts, which might be associated with CRS aggravation. These findings correspond well with those of earlier studies, which indicated that the PI3K/Akt and NF-κB signaling pathways play a crucial role in MMP-2 and TIMP-2 expression.

Although, steroids have severe side effects, they are still one of the most important drugs that can treat various diseases with anti-inflammatory and antioxidant properties. Steroids, such as dexamethasone and fluticasone propionate, have been successfully used in the therapy of various human inflammatory diseases, such as CRS. Steroids may also reduce mucosal inflammation and edema in paranasal sinuses and improve symptoms associated with CRS. However, recent evidence showed that glucocorticoids may affect not only the prevention of inflammation, but also the inhibition of tissue remodeling in CRS. It was shown that inhaled steroids inhibited TGF-β1-induced MMP expression in bronchial fibroblasts [33]. Steroids have been known to inhibit inflammation response and tissue remodeling by inhibiting various signaling pathway such as ROS and AKT signaling pathways [34,35]. Our study demonstrated that steroids down-regulated CSE-induced MMP-2 expression through inhibiting the ROS/PI3K/Akt and NF-κB signaling pathways. These results suggested that steroids contributed to the inhibition of MMP-2 expression in nasal fibroblasts.

Our study has some limitations. CRS is a multifactorial disorder, and its pathogenesis involves interactions between environmental insults, infectious loading, and genetic predisposition, it is not possible to directly translate the obtained results into the CRS model. However, indirectly, it may be thought that the regulation of MMP may influence tissue remodeling and thus contribute to the development of CRS. In addition to, we did not check the effect of CSE in CRS mouse model. We will check whether the exposure of CSE can aggravate symptoms of CRS.

## 5. Conclusions

We have provided here the first evidence that CSE increases MMP-2 production and inhibits TIMP-2 expression in nasal fibroblasts. However, CSE did not affect MMP-9 and TIMP-1 expression in nasal fibroblasts. Additionally, our study demonstrated the role of the ROS/PI3K/Akt and NF-κB signaling pathways in mediating the CSE-regulated MMP-2/TIMP-2 imbalance in nasal fibroblasts, which might contribute to tissue remodeling in CRS. The present study did not demonstrate the effect of CSE on MMP and TIMP expression, which was only examined in one type of submerged cell culture. Additional experiments are needed to clarify the results of the present study.

## Figures and Tables

**Table 1:** Sequences of real-time PCR oligonucleotide primers.

Primer		Sequence
*MMP-2*	Forward	5’- AGA TCT TCT TCT TCA AGG AAC CGT T -3’
	Reverse	5’- GGC TGG TCA GTG GCT TGG GGT A -3’
*MMP-9*	Forward	5’- GCG GAG ATT GGG AAC CAG CTG TA -3’
	Reverse	5’- GAC GCG CCT GTG TAC ACC CAC A -3’
*TIMP-1*	Forward	5’- ACC ACC TTA TAC CAG CGT TAT GA -3’
	Reverse	5’- GGT GTA GAC GAA CCG GAT GTC -3’
*TIMP-2*	Forward	5’- GCT GCG AGT GCA AGA TCA C -3’
	Reverse	5’- TGG TGC CCG TTG ATG TTC TTC
*TLR4*	Forward	5’- TGA GCA GTC GTG CTG GTA TC -3’
	Reverse	5’- CAG GGC TTT TCT GAG TCG TC -3’
